# Educational Needs and Priorities for Pediatric Emergency Nursing: A Cross-Sectional Study of Clinical Nurses

**DOI:** 10.3390/children13040501

**Published:** 2026-04-02

**Authors:** Jung Hwa Lee, So Yeon Park, Hyeon Ok Ju

**Affiliations:** 1 College of Nursing, Choonhae College of Health Sciences, Ulsan 44965, Republic of Korea; ljh87@ch.ac.kr; 2 College of Nursing, Dong-A University, Busan 49201, Republic of Korea; enfanju@dau.ac.kr

**Keywords:** emergency nursing, needs assessment, pediatric nursing, competence education, nursing

## Abstract

**Highlights:**

**What are the main findings?**

- Educational priorities in pediatric emergency nursing were concentrated in time-critical, high-risk conditions (e.g., status epilepticus, anaphylaxis, asthmatic attack, diabetic ketoacidosis, and meningitis).
- High priority skill gaps involved advanced, high-stakes procedures, especially airway/ventilator-related competencies (e.g., intubation preparation and ventilator operation/mode-setting) and immediate lifesaving interventions (e.g., CPR/defibrillation, high-risk medication and sedation care).

**What are the implications of the main findings?**

- Educational needs remained high across demographic and experience groups; therefore, curricula should implement continuous, standardized training rather than assuming competence will increase sufficiently with clinical experience alone.
- Simulation-based and mobile-enabled, scenario-driven modules should be prioritized to prepare students for rapid clinical deterioration and complex procedures that are difficult to practice consistently in real clinical placements.

**Abstract:**

Background/Objectives: Pediatric emergency nursing requires timely, accurate interventions, yet educational content is not always aligned with clinical priorities. Identifying and prioritizing educational gaps based on clinical relevance and nurses’ current performance is essential to improve pediatric emergency care. Methods: This descriptive cross-sectional study assessed clinical performance and educational needs among nurses working in emergency departments, general wards, and intensive care units. Data were collected using a structured questionnaire on 20 pediatric emergency conditions and related procedures. Priorities were identified using the Borich Needs Assessment and the Locus for Focus model, based on differences between required and present competence and the level of perceived importance. Results: Educational needs were consistently high across participant characteristics. In both the Borich needs assessment and the Locus for Focus model, the highest priorities were identified in pediatric emergency nursing competencies related to time-critical emergencies and core procedures, particularly resuscitation and high-risk medication administration. Conclusions: Educational priorities in pediatric emergency nursing span urgent conditions and skill-intensive procedures. Although performance varied by age and experience, educational needs were consistently high, supporting continuous, standardized training. Simulation-based and mobile-enabled, scenario-focused education should be considered to enhance preparedness and response capacity among nursing students and early-career nurses.

## 1. Introduction

Emergency medical care provides timely and appropriate treatment to critically ill patients and supports their recovery through coordinated follow-up [1]. In Korea, the emergency medical system has shown quantitative growth through the specialization of personnel, advancements in facilities and equipment, and the securing of financial resources and institutional support [2]. As of 2023, emergency room (ER) visits totaled 7,961,677, equivalent to 155.1 visits per 1000 individuals [3]. Excluding the period of reduced ER use during the COVID-19 pandemic, more than 7 million people have visited ERs annually [3].

Despite a decline in birth rates, ER utilization among children under 12 years increased, from 4.8 cases per 100 children in 2017 to 11.6 cases in 2022 [4]. Due to developmental limitations, children often cannot recognize or clearly express their symptoms, making assessment in pediatric emergency situations particularly challenging [5]. In some cases, guardian involvement may assist communication and information gathering during the care process [6]. This makes assessment in pediatric emergency cases particularly difficult [5]. Therefore, collaboration with guardians is essential to obtain necessary information and deliver effective care [6]. Given that vital signs, motor abilities, and cognitive development vary substantially by age, assessments must be tailored to both age and developmental stage [5].

Without timely and appropriate emergency care, children may develop serious complications or long-term sequelae. Careful observation and prompt, active intervention are thus essential [7]. Currently, only 12 pediatric emergency medical centers operate nationwide in Korea [8], resulting in marked regional disparities in access [4]. In addition, pediatric emergencies may arise not only in specialized centers but also in general wards or other non-specialized clinical settings, and children’s conditions can deteriorate rapidly. However, most ERs function as general emergency medical institutions for both adult and pediatric patients, limiting the provision of care tailored to children’s physiological and developmental characteristics [9].

Within this context of limited infrastructure, nurses who initially assess pediatric emergency patients play a critical role in determining patient outcomes through timely judgment and intervention [10]. To fulfill this role, they must demonstrate professional competence in pediatric-specific areas, including medication dosing, age-appropriate vital sign assessment, and effective communication with guardians [10,11,12]. In this context, clinical practicums in nursing education are intended to equip students with the ability to perform nursing skills and develop care plans in preparation for real-world clinical situations [13], serving as a foundation for the acquisition of core competencies needed in professional practice [14]. However, pediatric nursing practicums primarily involve hospitalized children or newborns, and due to infection risk and guardian presence, opportunities for direct clinical engagement are limited [15]. In addition, the lack of clinical cases, restricted availability of practice spaces, and shortage of practicum hospitals hinder adequate hands-on experience [15,16].

Pediatric emergency nursing requires a high level of expertise and rapid clinical judgment, and the importance of specialized education in this area has been consistently emphasized. However, relatively few studies have systematically examined which content should be prioritized in pediatric emergency nursing education. International studies have explored barriers to education and training needs related to pediatric trauma care among emergency personnel, identifying lack of guidelines, limited time, and insufficient resources as major obstacles to participation in training [17,18]. At the same time, healthcare professionals, including nurses and physicians, have recognized the need for pediatric emergency training and viewed simulation-based education as an effective approach [18]. Nurses caring for children have also reported an ongoing need for continuing education, particularly in trauma- and injury-related situations [19].

Nevertheless, these previous studies have mainly focused on barriers to education, the need for continuing education, or the effects of specific educational methods, and have provided limited evidence on what content should be prioritized across the broader domain of pediatric emergency nursing. A recent scoping review of educational interventions for emergency nurses similarly showed that educational programs can improve clinical practice behaviors, but also suggested that the evidence base remains limited in guiding which competencies need the most urgent educational attention in actual clinical settings [20]. This suggests that demonstrating the effectiveness of an educational method alone is not sufficient; evidence is also needed to determine what should be taught first.

In Korea, Jeon and Im [21] examined the frequency, importance, and competency levels of pediatric emergency nursing practice among emergency department nurses, and Yoo [22] developed and evaluated a simulation-based program for neonatal emergencies. Although these studies are meaningful in that they describe current practice or evaluate specific interventions, they do not fully address the prioritization of educational needs based on the gap between the level of competence required in clinical practice and nurses’ current competence. Because knowledge acquisition alone is insufficient in clinical settings, educational programs need to be designed in ways that reflect actual clinical demands [23]. Therefore, there is a need to systematically analyze the educational needs of pediatric emergency nurses in domestic clinical settings and to identify priorities for practice-based education.

In this study, we examined nurses’ experiences with pediatric emergency care and their educational needs across emergency rooms, general wards, and intensive care units. By drawing on the perspectives of nurses with clinical experience, we sought to identify educational content that should be emphasized for nursing students before clinical practice. This study was intended to provide baseline data for planning practice-based education in pediatric emergency nursing.

## 2. Materials and Methods

### 2.1. Design

This was a descriptive cross-sectional study of clinical nurses’ perceived educational needs and priorities in pediatric emergency nursing. By identifying the competencies required in current clinical settings, the study aimed to provide baseline data for the development of a mobile app-based educational program for nursing students. This study is reported in accordance with the Consensus-based checklist for reporting of survey studies (CROSS) guideline [24].

### 2.2. Participants

Participants were nurses working in ERs, general wards, or intensive care units (ICU) who were involved in pediatric emergency care at nine secondary and tertiary hospitals located in B metropolitan city, U metropolitan city, and the Gyeongnam and Gyeongbuk regions. In this study, pediatric emergency care referred to the initial assessment and nursing response to acute or potentially life-threatening conditions in pediatric patients. General ward and ICU nurses were included because pediatric emergencies may also occur in inpatient settings, where ward nurses are often responsible for early recognition and initial response. Inclusion criteria comprised understanding the study purpose, at least one year of experience in the relevant unit, and voluntary agreement to participate.

The sample size was calculated using G*Power 3.1.9 based on a previous study by Park [25]. For a paired *t*-test comparing required and present competence in pediatric emergency nursing, an effect size of 0.3, a significance level of 0.05, and a power of 0.95 were applied, yielding a minimum required sample size of 134. Considering a possible 20% dropout or non-response rate, 220 questionnaires were distributed. Of these, 215 completed responses were included in the final analysis.

### 2.3. Instruments

#### 2.3.1. General Characteristics

General characteristics included nine items: sex, age, educational level, type of medical institution, work pattern, position, current department, total clinical experience, and clinical experience related to pediatric care.

#### 2.3.2. Performance and Importance of Pediatric Emergency Nursing Care and Skills

Items assessing performance and importance were developed based on core pediatric emergency care requirements outlined by the Korean Society of Emergency Medicine [26] and relevant literature [7,27]. Initially, ten items related to nursing care for pediatric emergency conditions and 17 items related to pediatric emergency nursing skills were selected.

Content validity was evaluated by a panel of experts comprising four pediatric nursing professors with extensive emergency care experience and six clinical specialists. Each expert rated the items on a 4-point scale from 1 (very inappropriate) to 4 (very appropriate) and provided suggestions for additions, revisions, or deletions as needed. Based on these comments, the preliminary items were reviewed for clarity, relevance, and applicability to pediatric emergency nursing practice.

The Content Validity Index (CVI) was calculated according to criteria proposed by Polit and Beck [28]. Acceptance criteria were set at an Item-CVI of ≥0.78 and a Scale-CVI/Average (S-CVI/Ave) of ≥0.90. For the ten nursing care items related to pediatric emergency conditions, all items had an Item-CVI of ≥0.80, and the S-CVI/Ave was 0.94; thus, all items were retained. For the 17 pediatric emergency nursing skill items, three items had an Item-CVI of 0.70, while the remaining 14 items met criteria with an Item-CVI of ≥0.90. The overall S-CVI/Ave was 0.92. The three items with low scores: “lumbar puncture nursing,” “chest tube management nursing,” and “central venous catheter management nursing,” were noted as rarely performed in clinical practice. Following researcher discussion, these three items were removed, revising the final list of pediatric emergency nursing skill items to 14.

A 5-point Likert scale ranging from 5 (very high) to 1 (very low) was used to evaluate the level of performance in pediatric emergency nursing care and skills. To assess perceived importance, a separate 5-point Likert scale ranging from 5 (very important) to 1 (not important at all) was applied. Higher scores indicated a higher level of performance or greater perceived importance for each condition or skill.

Reliability of the instrument in this study was verified as follows: Cronbach α was 0.94 for performance and 0.95 for importance in pediatric emergency nursing care. For pediatric emergency nursing skills, Cronbach α was 0.95 for performance and 0.97 for importance. The final questionnaire items used in this study are provided in Supplementary S1.

### 2.4. Data Collection and Ethical Considerations

This study was approved by the Institutional Review Board (IRB) of the affiliated institution (IRB No. 1044386-A-2024-005) and conducted in accordance with the principles of the Declaration of Helsinki. From November to December 2024, nurses working in ERs, general wards, and intensive care units involved in pediatric emergency care were recruited from nine secondary and tertiary hospitals located in B metropolitan city, U metropolitan city, and the Gyeongnam and Gyeongbuk regions. Participants were eligible if they understood the purpose of the study, had at least one year of work experience in the relevant department, and voluntarily agreed to participate.

The researcher explained the purpose and procedures of the study to the nursing departments of each institution and obtained prior approval. A recruitment notice was posted, and nurses wishing to participate were provided a study information sheet detailing study purpose, eligibility, procedures, potential risks and benefits, privacy protection measures, consent and withdrawal procedures, and plans for use of the study results. Only those who voluntarily agreed to participate completed the questionnaire.

The survey was conducted both online and offline. Eligible nurses at the participating hospitals received study information and were invited to complete the questionnaire either online through a QR code-linked survey or offline using a printed form. Paper questionnaire were distributed with sealed return envelopes so that completed surveys could be returned confidentially. Access to the online survey was limited to those who received the survey link, and the questionnaire was programmed to begin only after participants selected “Yes” to indicate consent. For the paper-based survey, informed consent was obtained prior to questionnaire completion. Participants were instructed to respond only once, and all returned questionnaires were reviewed for potential duplicate responses before analysis. The questionnaire required approximately 10–15 min to complete.

### 2.5. Data Analysis

Data were analyzed using IBM SPSS Statistics version 29.0. Descriptive statistics were used to summarize participants’ general characteristics. A paired *t*-test was conducted to compare the required competence level (RCL) and the present competence level (PCL) in pediatric emergency nursing. Differences according to general characteristics were analyzed using independent *t*-tests and one-way ANOVA with Scheffé’s test for post hoc comparisons.

Educational priorities were identified using Borich’s needs assessment formula [29] and the Locus for Focus model [30]. Borich need scores were calculated by weighting the discrepancy between the RCL and PCL by the mean RCL and dividing it by the total number of cases. In the Locus for Focus model, items located in the first quadrant (high importance and high discrepancy) were considered the highest priority for education. Items ranked highly in both analyses were classified as top or secondary priorities for educational interventions.

## 3. Results

### 3.1. General Characteristics of the Participants

A total of 220 questionnaires were distributed to eligible nurses involved in pediatric emergency care across the nine participating hospitals. Two hundred fifteen questionnaires were returned, yielding a response rate of 97.7%, and all 215 responses were included in the final analysis. The general characteristics of the participants are summarized in Table 1. Among the nurses included in the study, the largest proportion were in their 20s (38.60%). A four-year university degree was the most common educational background (62.79%). Most participants were employed in general hospitals (70.70%), and the majority held staff nurse positions (72.09%). With respect to pediatric nursing experience, 59.07% had 1 to 3 years of experience (Table 1).

### 3.2. Differences in Current Competence and Educational Need Scores for Pediatric Emergency Conditions and Nursing Skills According to General Characteristics

As shown in Table 1, current competence for pediatric emergency conditions and symptoms differed significantly according to educational level (F = 3.04, *p* = 0.050) and pediatric nursing experience (F = 3.72, *p* = 0.012). Nurses with higher educational attainment and more pediatric nursing experience reported higher levels of current competence. In contrast, Borich need scores for pediatric emergency conditions and symptoms did not differ significantly according to general characteristics. Current competence for pediatric emergency nursing skills also differed significantly by age (F = 3.87, *p* = 0.010), with older nurses reporting higher levels of performance. However, Borich need scores for pediatric emergency nursing skills did not differ significantly according to general characteristics (Table 1).

### 3.3. Educational Needs Analysis Using the Borich Model for Pediatric Emergency Conditions and Nursing Skills

A paired *t*-test was performed to assess differences between the required competence level and the current competence level for pediatric emergency conditions and nursing skills. Statistically significant differences were observed for all items (*p* < 0.001) (Table 2). Based on the results of the Borich needs assessment, status epilepticus showed the highest educational need score (5.82) among pediatric emergency conditions, followed by anaphylaxis, asthmatic attack, diabetic ketoacidosis, and meningitis.

Among pediatric emergency nursing skills, “setting ventilator modes” had the highest educational need score (6.54), followed by “operating a ventilator,” “preparing for pediatric endotracheal intubation,” “nursing care related to ventilator use,” “maintenance care for endotracheal intubation,” and “cardiopulmonary resuscitation (chest compression, defibrillation)” (Table 2).

### 3.4. Locus for Focus Model for Pediatric Emergency Conditions and Nursing Skills

**Figure 1:**
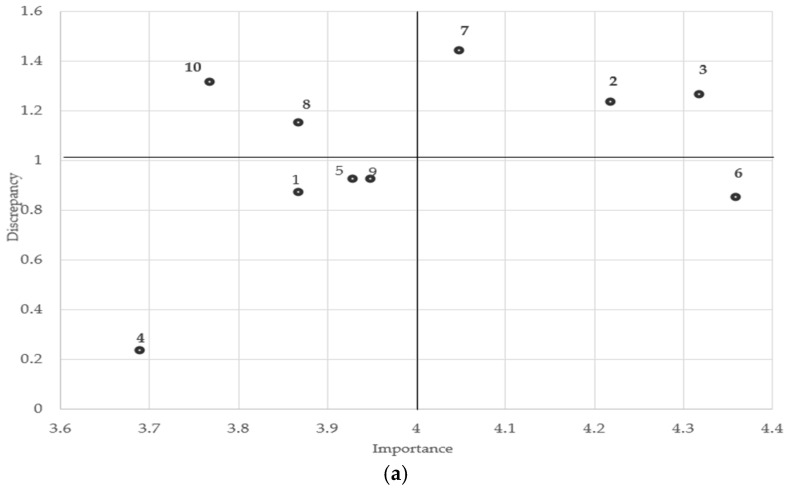

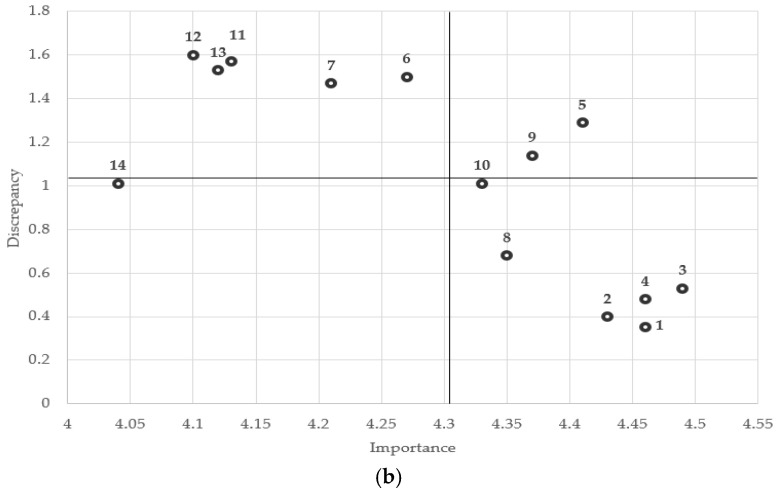
(**a**) Locus for Focus model identifying educational priorities for pediatric emergency conditions. 1, acute epiglottitis; 2, asthmatic attack; 3, anaphylaxis; 4, acute gastroenteritis; 5, intussusception; 6, febrile seizure; 7, status epilepticus; 8, meningitis; 9, hypoglycemia; 10, diabetic ketoacidosis. (**b**) Locus for Focus model identifying educational priorities for pediatric emergency nursing skills. 1, vital sign assessment; 2, pulse oximetry connection and interpretation; 3, assessment of consciousness; 4, assessment of respiratory distress; 5, cardiopulmonary resuscitation and defibrillation; 6, preparation for pediatric endotracheal intubation; 7, nursing care for pediatric endotracheal tube maintenance; 8, administration of oxygen therapy; 9, nursing care related to high-risk medications; 10, nursing care related to sedative administration; 11, operation of mechanical ventilator; 12, setting ventilator modes; 13, nursing care for patients on ventilator; 14, blood transfusion.

The priority levels of nurses’ educational needs for pediatric emergency conditions and nursing skills were visualized using the Locus for Focus model, as presented in Figure 1a,b. Conditions positioned in the first quadrant (High Importance–High Discrepancy), where both the importance level and the gap between importance and performance exceeded the average, included anaphylaxis, asthmatic attacks, and status epilepticus. For pediatric emergency nursing skills, the first quadrant included “cardiopulmonary resuscitation (chest compression, defibrillation),” “nursing care related to high-risk medications” (Figure 1a,b).

## 4. Discussion

Prompt nursing interventions in emergency settings are directly associated with the recovery and survival of pediatric patients, underscoring the importance of enhancing the competencies of skilled nurses in critical care environments [31]. To be effective, nursing education must extend beyond fragmented technical instruction and incorporate a comprehensive understanding of pediatric conditions, along with structured, skill-based training [18,23].

Accordingly, we analyzed performance ability, educational needs related to pediatric emergency conditions, and the nursing skills of clinical nurses involved in pediatric emergency care across ERs, general wards, and intensive care units. Using Borich’s needs assessment and the Locus for Focus model, we identified educational priorities and proposed directions for pediatric emergency nursing education targeted at nursing students.

Performance ability related to pediatric emergency conditions and nursing skills differed based on general characteristics such as educational level, clinical experience, and age. Notably, higher performance levels were observed among nurses with greater pediatric nursing experience and older age, likely reflecting the impact of accumulated clinical exposure, which enhances proficiency in emergency situations [32] and supports the development of clinical judgment through experience with diverse cases [33].

In contrast, educational needs did not vary significantly across general characteristics, indicating that the perceived necessity for pediatric emergency nursing education remains high irrespective of background. This finding aligns with Lehmann et al. [34], who reported that nurses acknowledged the need for training in core skills and protocols for managing common pediatric emergencies. Given the demands of pediatric emergencies, characterized by the need for rapid decision-making and technical precision, even experienced nurses require regular training. These findings reinforce the need for a sustainable and continuous educational framework for pediatric emergency nursing [17].

According to the Borich Needs Assessment, the items identified as having the highest educational needs were high-risk, life-threatening conditions, including status epilepticus, anaphylaxis, asthmatic attack, diabetic ketoacidosis, and meningitis. This finding is consistent with previous studies [5,10], which reported that children are more vulnerable to rapid clinical deterioration and that the appropriateness of initial nursing interventions has a critical impact on patient outcomes.

Emergency conditions such as status epilepticus, anaphylaxis, asthmatic attack, diabetic ketoacidosis, and meningitis, which are commonly encountered in pediatric care, are highly time-sensitive and demand prompt assessment and intervention [35]. Accordingly, clinical expertise and sound decision-making are essential in managing such cases.

In the assessment of educational needs for nursing skills, procedures requiring advanced technical proficiency—such as “setting ventilator modes,” “preparing for endotracheal intubation,” and “cardiopulmonary resuscitation (CPR)”—were ranked among the highest. This observation is consistent with findings by Jeon and Im [21], who reported that these procedures were perceived by nurses as both frequently performed and highly important.

In the Locus for Focus model analysis, “cardiopulmonary resuscitation,” and “nursing care related to high-risk medications” were all located in the first quadrant, where both importance and performance discrepancy scores were high. This indicates that the ability to perform immediate interventions is directly associated with patient survival.

The slight differences in the top-priority items identified by the Borich needs assessment and the Locus for Focus model stem from the distinct analytical criteria of each method. The Borich formula generates a single composite score that is heavily influenced by the magnitude of the competency gap (the difference between required and current levels). Consequently, highly specialized skills with substantial competency gaps, such as “setting ventilator modes,” ranked at the top of the Borich assessment. In contrast, the Locus for Focus model requires an item to exceed the overall average thresholds for both importance (required level) and the competency gap to be placed in the first quadrant. Therefore, while “setting ventilator modes” presents a critical competency gap, its overall required frequency across all surveyed departments may have been slightly below the mean, excluding it from the first quadrant. Conversely, broadly applicable critical skills like “nursing care related to high-risk medications” met both above-average thresholds, demonstrating the value of using both methods complementarily to identify realistic educational priorities.

Burgess et al. [36] demonstrated that early intervention by skilled nurses significantly reduces treatment delays and contributes to symptom relief and decreased hospitalization rates in pediatric patients. A separate study similarly reported that education targeting clinical competency reduced time-to-treatment during emergencies, alleviated symptoms, and lowered hospitalization rates [36].

Similarly, the present study found that nurses perceived certain emergency conditions and procedures as highly important, yet rated their performance levels as comparatively low, particularly in scenarios requiring rapid intervention.

This study found clear gaps between nurses’ self-rated performance and the level of competence they believed was required for specific pediatric emergency tasks. Pediatric emergencies, in particular, require multidimensional competencies, including accurate medication dosage calculation, interpretation of vital signs, and effective communication with caregivers [20]. However, due to structural constraints, nursing students often encounter limited clinical exposure and have few opportunities to manage emergency scenarios directly [15,16].

To bridge this gap, it is essential to implement simulation-based and mobile learning strategies that prepare nursing students for pediatric emergencies prior to entering clinical practice. In addition, simulation-based approaches such as telesimulation may be particularly useful for reinforcing pediatric emergency competencies when direct clinical exposure is limited or uneven across practice settings [9,20,22].

A strength of this study is that it identified educational priorities based on clinical nurses’ experiences. These findings may be useful when designing pediatric emergency nursing curricula. Consistent with the findings of Roney and Acri [19], who highlighted a strong need for ongoing education among pediatric emergency nurses, such evidence-based assessments can promote engagement in educational programs and optimize learning outcomes [22].

Using both the Borich needs assessment and the Locus for Focus model allowed us to examine educational priorities from two complementary perspectives [37]. This helped identify topics that were not only important but also showed meaningful gaps between required and present competence.

These findings also have implications for both undergraduate nursing curricula and continuing education in healthcare institutions. Because not all pediatric emergency topics can be covered with equal emphasis, educational content may need to be prioritized according to clinical importance and the gap between required and current competence. In this context, simulation-based education may be particularly useful for high-priority items, as it allows repeated practice of rapid clinical judgment, immediate intervention, and safe performance in high-risk situations [19,20,22].

Based on these findings, pediatric emergency content in nursing education may need to place greater emphasis on high-risk conditions and core emergency skills. Simulation-based and scenario-based learning may be particularly useful for helping students prepare for these situations before entering clinical practice.

This study has several limitations. First, because participants were recruited from specific geographic regions, the findings may not be fully generalizable to other settings with different healthcare systems or nursing education structures. Differences in pediatric emergency service structures, clinical exposure opportunities, and educational environments across institutions or countries may also influence the applicability of the findings. Second, since the data were collected using self-reported questionnaires, the study assessed perceived rather than actual clinical competence. As a result, participants may have overestimated or underestimated their own performance. In addition, because data were collected using both online and paper-based questionnaires, potential differences according to the mode of administration cannot be completely excluded. Third, although the educational topics were selected based on previous literature and clinical experience, they may not reflect the full range of pediatric emergency situations encountered in practice.

## 5. Conclusions

This study examined the performance ability and educational needs of nurses involved in pediatric emergency care, focusing on emergency conditions and core nursing skills. Educational priorities were identified using Borich’s needs assessment and the Locus for Focus model. The results indicated that life-threatening conditions and nursing skills requiring a high level of proficiency were the top educational priorities. Differences in performance ability were observed based on clinical experience and age, while educational needs remained high regardless of general characteristics. These findings underscore the need for continuous and standardized education in pediatric emergency care.

Based on these findings, nursing education institutions should enhance content that reflects clinical demands and adopt simulation-based and mobile instructional strategies incorporating scenario-based, practice-oriented learning. Furthermore, offering practice opportunities that align with real clinical needs can improve nursing students’ emergency response capabilities and contribute to better pediatric outcomes and overall care quality.

## Figures and Tables

**Table 1:** Differences in Nursing Competence and Required Competence of Pediatric emergencies and Pediatric Nursing Skills (N = 215).

Variables	Categories	N	(%)	PCL				RCL			
				Pediatric Emergencies		Pediatric Nursing Skill		Pediatric Emergencies		Pediatric Nursing Skill	
				M ± SD	t or F (*p*)	M ± SD	t or F (*p*)	M ± SD	t or F (*p*)	M ± SD	t or F (*p*)
Age(years)	20~29	83	(38.60)	2.91 ± 0.90	0.402 (0.752)	3.21 ± 0.71	3.879 (0.010)	4.10 ± 0.74	0.769 (0.513)	4.38 ± 0.79	2.117 (0.099)
	30~39	78	(36.28)	3.05 ± 0.88		3.37 ± 0.91		3.94 ± 0.82		4.36 ± 0.73	
	40~49	44	(20.47)	2.95 ± 0.95		2.98 ± 0.96		3.91 ± 0.97		4.03 ± 0.99	
	<50	10	(4.65)	3.13 ± 0.93		3.88 ± 0.89		3.96 ± 0.79		4.15 ± 0.84	
Education level	College	53	(24.65)	2.81 ± 0.78	3.048 (0.050)	3.20 ± 0.80	1.078 (0.342)	4.00 ± 0.78	0.002 (0.998)	4.38 ± 0.80	0.637 (0.530)
	University	135	(62.79)	2.98 ± 0.90		3.22 ± 0.85		3.99 ± 0.84		4.28 ± 0.81	
	Master’s or higher	27	(12.56)	3.34 ± 1.06		3.48 ± 1.03		4.01 ± 0.87		4.16 ± 0.96	
Hospital type	Primary	5	(2.33)	2.64 ± 1.54	2.709 (0.069)	3.45 ± 1.33	2.476 (0.087)	4.18 ± 1.13	1.849 (0.160)	4.51 ± 0.86	0.417 (0.659)
	Secondary	152	(70.70)	2.91 ± 0.81		3.17 ± 0.79		4.06 ± 0.76		4.31 ± 0.75	
	Tertiary	58	(26.98)	3.21 ± 1.03		3.45 ± 0.98		3.82 ± 0.94		4.22 ± 1.00	
Nurse position	Registered	155	(72.09)	2.92 ± 0.85	1.529 (0.156)	3.23 ± 0.82	0.813 (0.445)	3.97 ± 0.81	2.857 (0.060)	4.30 ± 0.82	0.695 (0.500)
	Charge	29	(13.49)	3.03 ± 0.94		3.16 ± 0.97		3.82 ± 0.88		4.14 ± 0.94	
	Head	31	(14.42)	3.26 ± 1.07		3.43 ± 0.98		4.30 ± 0.77		4.39 ± 0.73	
Working unit	ER	50	(23.25)	2.99 ± 1.11	0.040 (0.961)	3.27 ± 1.10	0.020 (0.980)	4.01 ± 0.97	0.010 (0.990)	4.31 ± 0.92	0.010 (0.990)
	ICU	23	(10.69)	2.98 ± 1.11		3.26 ± 1.10		4.00 ± 0.98		4.30 ± 0.93	
	GW	142	(66.06)	2.96 ± 1.09		3.24 ± 1.10		3.99 ± 0.97		4.29 ± 0.93	
Pediatric clinical experience(years)	1~<3	127	(59.07)	2.81 ± 0.80	4.347 (0.002)	3.19 ± 0.77	750 (0.523)	4.00 ± 0.83	1.163 (0.328)	4.37 ± 0.76	2.220 (0.087)
	3~<7	30	(13.95)	3.29 ± 1.09		3.22 ± 1.09		3.85 ± 0.81		3.99 ± 1.02	
	7~<10	24	(11.16)	3.21 ± 0.99		3.33 ± 0.86		4.20 ± 0.67		4.41 ± 0.60	
	≥10	34	(15.81)	3.17 ± 0.90		3.43 ± 0.98		3.91 ± 0.90		4.17 ± 0.95	

ER = Emergency department; GW = General ward; ICU = Intensive care unit; M = mean; PCL = present competence level; RCL = required competence level; SD = standard deviation.

**Table 2:** The Borich Needs for Pediatric Emergency Nursing Competence and Clinical Skills (N = 215).

Variables	Categories	Differences				Borich Needs	Borich’s Priorities	Locus for Focus Model’s Priorities
		PCL	RCL	RCL-PCL	t (*p*)			
		M ± SD	M ± SD	Gap				
Pediatric emergencies	Acute epiglottitis	3.00 ± 1.11	3.87 ± 0.99	0.87	−11.31 (<0.001)	3.37	9	
	Asthmatic attack	2.99 ± 1.03	4.22 ± 0.91	1.23	−15.96 (<0.001)	5.20	3	○
	Anaphylaxis	3.06 ± 1.09	4.32 ± 0.89	1.26	−16.31 (<0.001)	5.42	2	○
	Acute gastroenteritis	3.47 ± 1.07	3.69 ± 0.97	0.23	10.90 (<0.001)	0.84	10	
	Intussusception	3.01 ± 1.13	3.93 ± 0.97	0.92	−11.53 (<0.001)	3.60	8	
	Febrile seizure	3.51 ± 1.14	4.36 ± 0.90	0.85	−11.53 (<0.001)	3.69	6	
	Status epilepticus	2.61 ± 1.09	4.05 ± 1.00	1.44	−17.43 (<0.001)	5.82	1	○
	Meningitis	2.71 ± 1.12	3.87 ± 0.98	1.15	−14.33 (<0.001)	4.46	5	
	Hypoglycemia	3.03 ± 1.19	3.95 ± 1.04	0.92	−11.61 (<0.001)	3.62	7	
	Diabetic ketoacidosis	2.46 ± 1.09	3.77 ± 1.06	1.31	−15.00 (<0.001)	4.92	4	
Pediatric Nursing Skills	Vital sign	4.11 ± 0.90	4.46 ± 0.78	0.35	7.17 (<0.001)	1.55	14	
	Pulse oximetry connection and interpretation	4.04 ± 0.93	4.43 ± 0.79	0.40	7.63 (<0.001)	1.75	13	
	Assessment of consciousness	3.96 ± 0.98	4.49 ± 0.76	0.53	10.49 (<0.001)	2.38	11	
	Assessment of respiratory distress	3.98 ± 0.94	4.46 ± 0.81	0.48	9.32 (<0.001)	2.14	12	
	Cardiopulmonary resuscitation and defibrillation	3.13 ± 1.07	4.41 ± 0.90	1.29	18.38 (<0.001)	5.69	6	○
	Preparation for pediatric endotracheal intubation	2.77 ± 1.15	4.27 ± 0.92	1.50	19.21 (<0.001)	6.39	3	
	Nursing care for pediatric endotracheal tube maintenance	2.73 ± 1.15	4.21 ± 0.95	1.47	18.85 (<0.001)	6.21	5	
	Administration of oxygen therapy	3.67 ± 1.17	4.62 ± 0.91	0.68	10.57 (<0.001)	2.97	10	
	Nursing care related to high-risk medications	3.22 ± 1.16	4.37 ± 0.93	1.14	15.70 (<0.001)	5.00	7	○
	Nursing care related to sedative administration	3.32 ± 1.13	4.33 ± 0.99	1.01	14.63 (<0.001)	4.39	8	
	Operation of mechanical ventilator	2.55 ± 1.17	4.13 ± 1.06	1.57	19.32 (<0.001)	6.49	2	
	Setting ventilator modes	2.51 ± 1.16	4.10 ± 1.05	1.60	19.70 (<0.001)	6.54	1	
	Nursing care for patients on ventilator	2.58 ± 1.17	4.12 ± 1.05	1.53	18.78 (<0.001)	6.32	4	
	Blood transfusion	3.02 ± 1.31	4.04 ± 1.16	1.01	12.75 (<0.001)	4.09	9	

M = mean; PCL = present competence level; RCL = required competence level; SD = standard deviation. In the Borich formula, N refers to the number of valid responses, which was 215 for all items.

## Data Availability

The datasets analyzed during the current study are available from the corresponding author on reasonable request. The data are not publicly available due to ethical restrictions and the protection of participants’ personal information.

## Supplementary Materials

The following supporting information can be downloaded at: https://www.mdpi.com/article/10.3390/children13040501/s1, S1: Final Questionnaire Items Used in This Study.
