# Supplementary material for: Educational Needs and Priorities for Pediatric Emergency Nursing: A Cross-Sectional Study of Clinical Nurses
Source: Children (Basel). 2026 Apr 2;13(4):501. doi: 10.3390/children13040501 (PMC13114470; doi:10.3390/children13040501)
Supplement: Supplementary file 1 [file children-13-00501-s001.zip › children-4193198-supplementary.pdf]

Supplementary S1. Final Questionnaire Items Used in This Study

Response scales used in the questionnaire

A. Present competence (PCL)
Each item was rated on a 5-point Likert scale: 1 = very low, 2 = somewhat low, 3 = moderate, 4 = somewhat high, 5 = very high.
B. Required competence (RCL)
Each item was rated on a 5-point Likert scale: 1 = not important at all, 2 = somewhat unimportant, 3 = moderate, 4 = important, 5 = very important.

Part 1. General characteristics
1. Age
2. Education level
3. Hospital type
4. Work type
5. Nurse position
6. Current department
7. Total nursing career
8. Pediatric-related clinical experience
9. Whether the participant had experienced pediatric emergency situations while working as a nurse
10. Types of pediatric emergency conditions/symptoms experienced
11. Frequency of experience with pediatric emergency nursing skills

Part 2. Pediatric emergency conditions
Participants rated both their PCL and RCL for the following 10 pediatric emergency conditions:
1. Acute epiglottitis
2. Asthmatic attack
3. Anaphylaxis
4. Acute gastroenteritis
5. Intussusception
6. Febrile seizure
7. Status epilepticus
8. Meningitis
9. Hypoglycemia
10. Diabetic ketoacidosis

Part 3. Pediatric emergency nursing skills
Participants rated both their PCL and RCL for the following 14 pediatric emergency nursing skills:
1. Vital sign measurement
2. Connection and interpretation of pulse oximetry monitoring
3. Assessment and observation of mental status
4. Assessment of dyspnea / respiratory difficulty
5. Cardiopulmonary resuscitation (chest compression, defibrillation)
6. Preparation for pediatric endotracheal intubation
7. Maintenance care for pediatric endotracheal intubation
8. Prescribed oxygen therapy
9. Nursing care related to high-risk medications
10. Nursing care related to sedative administration
11. Operating a ventilator

12. Setting ventilator modes
13. Nursing care related to ventilator use
14. Blood transfusion

Note. The final questionnaire consisted of general and experience-related characteristics, 10 pediatric emergency condition items, and 14 pediatric emergency nursing skill items. The emergency condition and nursing skill items were each rated for **present competence (PCL)** and **required competence (RCL)** using 5-point Likert scales.
